# Study on Technological Effects of a Precise Grooving of AlSi13MgCuNi Alloy with a Novel WCCo/PCD (DDCC) Inserts

**DOI:** 10.3390/ma13112467

**Published:** 2020-05-28

**Authors:** Szymon Wojciechowski, Rafał Talar, Paweł Zawadzki, Stanisław Legutko, Radosław Maruda, Chander Prakash

**Affiliations:** 1 Faculty of Mechanical Engineering, Poznan University of Technology, Piotrowo 3, 60-965 Poznan, Poland; rafal.talar@put.poznan.pl (R.T.); pawel.zawadzki@put.poznan.pl (P.Z.); stanislaw.legutko@put.poznan.pl (S.L.); 2 Faculty of Mechanical Engineering, University of Zielona Gora, 4 Prof. Z. Szafrana street, 65-516 Zielona Gora, Poland; r.maruda@ibem.uz.zgora.pl; 3 Department of Mechanical Engineering, University Institute of Engineering & Technology, Panjab University, Chandigarh 160014, India; chander.mechengg@gmail.com

**Keywords:** WCCo/PCD (DDDCC), machining, Al-Si alloy, tool wear, surface topography

## Abstract

The WCCo/PCD (Diamond Dispersed Cemented Carbide—DDCC) manufactured with the use of PPS (pulse plasma sintering) are modern materials intended for cutting tools with the benefits of tungsten carbides and polycrystalline diamonds. Nevertheless, the cutting performance of DDCC materials are currently not recognized. Thus this study proposes the evaluation of technological effects of a precise groove turning process of hard-to-cut AlSi13MgCuNi alloy with DDCC tools. The conducted studies involved the measurements of machined surface topographies after grooving with different cutting parameters. In addition, the tool life and wear tests of DDCC inserts were conducted during grooving process and the obtained results were compiled with values reached during machining with cemented carbide tools. It was also proved that grooving of AlSi13MgCuNi alloy with DDCC inserts enables 5 times longer tool life and almost 3-fold increase of cutting path compared to values obtained during grooving with H3 and H10 cemented carbide inserts. Ultimately, the feed value of *f* = 0.15 mm/rev and cutting speed in a range of 800 m/min ≤ *v_c_* ≤ 1000 m/min during grooving with DDCC inserts can be defined as an optimal machining parameters, enabling the maximization of tool life and improvement in surface quality.

## 1. Introduction

The aluminum alloys are widely applied in various industries due to their ability to combine strength and lightness [1]. In comparison with other construction materials, Al is characterized by a low melting temperature, in the range between 590 and 660 °C [2], excellent electrical [3] and thermal conductivity [4] and high processing ability by casting, stamping, drawing, spinning, rolling and hammering [5,6]. The mechanical properties, particularly hardness and strength, of these alloys can be improved by alloy additions such as copper, manganese, magnesium, zinc, silicon and others [1,7,8,9]. It is the basis for the fundamental classification of aluminum in functional terms [10]. The 2xxx (Cu addition), 7xxx (Sn) and 6xxx (Mg and Si) aluminum alloy classes are generally used for the automotive industry and massive loaded aviation constructions, as compressors inserts, ribs or fan discs working at temperatures of 200–300 °C [11]. The 3xxx (Mn) alloys are characterized by excellent thermal stability at high temperatures and retaining their mechanical properties [12]. This series of aluminum alloys are commonly employed in the manufacturing process of beverage cans [13].

In the case of 4xxx alloys series, which are generally used at temperatures lower than 230 °C, the primary alloying element is silicon (Si) [2,14]. Silicon is a common impurity in commercial aluminum alloys (0.01% to 0.15%) but is also used as a factor for reducing the cracking tendency in other alloys (0.5% to 4.0%) [2,15]. These alloys constitute the first group of Al-Si alloys. The next group of Al-Si alloys consists up to 12% Si content and is the most popular material for casting, cladding for brazing sheet [2], rods, wires for welding [16], as well as pistons of combustion engines [17,18]. The last group of aluminum-silicon alloys contains up to 23% of Si. These casting alloys are used for wear applications, for engine blocks [2] pistons, brake discs, brake drums, piston sleeves [1]. Besides, Al-Si alloys are applied widely in the casting industry because of thermal expansion coefficient at the low level, high strength-to-mass ratio and acceptable abrasion [19,20]. Alloys containing from 5% to 12% are characterized by high ductility and impact resistance, whereby are used in bridge railing supports [2]. As an addition to aluminum alloys, silicon impedes the kinetics of the chemical formation of the undesirable intermetallics [21]. Silicon is intentionally added to some alloys to avoid the dimensional instability problem [22] or, including magnesium, to keep precipitation hardening [2,23]. According to Pio et al. [24] and Jigajinni et al. [25], the mechanical properties of Al-Si alloys can also be enhanced by grain refinement using boron, titanium or strontium additions. Aluminum alloys have also high corrosion resistance; however, in case of aluminum-silicon, the effect of silicone particles induces an opposite effect on corrosion resistance due to its cathodic properties to the aluminum matrix [2,26]. However, the intensity of silicon content increases the eutectic fraction, which affects the increase of electrochemical corrosion [27].

Machining of Al-Si alloys is characterized by a large chip-tool contact area, excessive forces, process power and high temperatures in the cutting zone [1,28]. Moreover, the machining process indicates long and stringy chips [29] with a high thickness ratio [30], which results in low surface finishing [1]. Barzani et al. [31] observed that the build-up intensity decreases with an increase in silicon content, which is associated with changes in the plasticity of the Al-Si alloy. It was also noted that the intensity of adhesion during turning of the high-silicon alloy Al-11.3Si-2Cu with a cemented carbide inserts KU 10/TiN depends on the presence of certain alloying elements. The underlying mechanisms of tool wear during cutting of high-silicon Al-Si alloys are—abrasion as a result of scratching of the cutting tool, as well as microchipping of the cutting edge, associated with build-up-edge removal [32,33]. According to Kamiya et al. [34], the wear intensity of the K10 cemented carbide cutting insert increases with an increasing percentage content of silicon in the Al-Si alloy (and with the growth of cutting speed), which is caused by increasing the number of crushed silicon particles during cutting, affecting the tool abrasion intensity. Andrewes et al. [35] observed that a higher build-up-edge (BUE) occurs for a diamond-coated chemically vapour deposited (CVD) insert than in polycrystalline diamond (PCD) cutting tools. According to results, micro-unevenness and scratches on the surface of the applied diamond coating induce an increase in adhesion intensity.

During cutting of Al-Si alloy, hard particles such as silicon, acting as chip breakers, produce short chips, margin built-up-edge and excellent surface finish, although contribute to the decline in tool life [2]. During the cutting of aluminum alloys, forces can be lower, surface quality upgraded and flank wear decreased by application of sharp cutting edges (low values of cutting edge radii) and flank/rake faces of cutting tools with a low surface roughness [36,37]. The cutting forces are relatively low compared with ones reached during machining of steel but the energy required to cut is higher due to high cutting speeds and increases with hardness and mechanical strength [38]. Braga et al. [39] reported that cutting power grows with the increase of feed rates and machining length during drilling of Al alloy with 7 wt.% of Si. An increase in cutting temperature of aluminum alloys can cause microstructural transformation, high residual stresses in the machined surface, geometrical errors of a machined parts and can also increase the alloy ductility [40,41,42,43]. The highest values of temperatures during machining were observed in the machining of workable or cast alloys [44,45]. In the case of silicon contents, the machining temperature can reach from 350 to 750 °C [46] which can indicate increased flank wear and higher stresses on the machined surfaces [44]. The high thermal conductivity of Al provide quick heat spread across the whole workpiece, which results in thermal expansion of the fixture system. One of the most critical machining factors is surface integrity [47]. The surface roughness is mainly dependent on alloy hardness and microstructural characteristics [48]. The higher the hardness, the lower the surface roughness, because the material adhesion to the cutting edge is reduced [11]. Additionally, low chip breakability causes damage to surface integrity [49]. However, according to Manna et al. [50], in the case of hard particle additions, such as Si, build-up-edge and pullout of hard particles may occur. Moreover, the affinity of aluminum alloys with cutting tools materials such as TiC, TiN or Al_2_O_3_ induces material accumulation on the cutting tool. These phenomena result in deterioration of the surface quality during cutting in different cooling/lubricating conditions [51,52]. According to Kamiya et al. [53] the appearance of Si in the Al-Si alloy has a significant influence on surface roughness. Growth of the silicon content in the Al-Si alloy, in the range of 2% ≤ Si ≤ 12% significantly reduced the value of the surface roughness parameter *Ra*. Furthermore, Barzani et al. [31] confirmed these results and showed that for most of the set values of cutting speed and feed, the smallest values of surface roughness *Ra* occurred during machining of the Al-Si alloy containing bismuth. Also, Kuczmaszewski et al. [54] showed that the surface topography is dependent on the cutting speed, the type of tool material and the condition of the insert. Besides, another problem is thermal expansion, which is strictly connected with cutting temperature during milling of aircraft and engine parts. It can also depend on the geometry and properties of workpiece, the tool and kinematics of machining operation [1].

High-speed steels, sintered carbides and diamond tools are the most popular in the machining of Al alloys, because of their low chemical affinity for aluminum and reducing the adherent layer on the tool. In case of turning processes of aluminum alloys with a low Si content, tungsten carbide K10 grade inserts are usually recommended [55]. The K20 grade inserts are implemented during machining with rapid temperature variations and K01 is employed for machining with the use of abrasive particles. Cemented carbide tools are very often applied in the turning of soft aluminum alloys with high cutting speeds (600–800 m/min), as well as with positive rake angles (6°–20°). This information was confirmed during the research conducted by Torpov et al. [55]. During cutting of Al-Si alloys with a Si content in a range between 12–15 vol.%, the rake angle values from 0° to 7° are recommended, independently of the type of insert material [1]. Referring to Manna et al. [50], the cutting speed up to 225 m/min for cutting of Al/SiC with uncoated cemented carbide is approved; also, Ciftci et al. [56] suggested the use of cutting speeds in the range between 20 and 80 m/min. Moreover, Kamiya et al. [53] recommended *v_c_* values from 20 to 450 m/min for Al/SiC materials. However, in case of Al-Si alloys with high Si content and high cutting speeds, (>600 m/min) [55] the diamond-based and polycrystalline diamond tools are suggested for machining [36,57]. It should be noted that ceramic cutting tools containing nitrides should not be applied during cutting of aluminum alloys due to intensive adhesion and the formation of BUE [41,58,59]. Itoigawa et al. [60] compared a machining performance of PCD and cemented carbide tools during machining of AlSi5 alloy. By setting the cutting speed within the range from 200 to 800 m/min, it has been presented that specific force for the diamond tool is smaller than for carbide insert. More comprehensive comparative studies of dry aluminum turning were conducted by Chattopadhyay et al. [61]. Researchers compared K10 tungsten carbide (WC) insert (three types), with a tools with different coatings (CVD TiC WC, CVD TiN WC, CVD Al_2_O_3_ WC and HFCVD WC). The authors concluded that the application of HFCVD inserts during cutting caused the lowest degradation degree of a tool, resulting from a low chemical affinity with Al alloy. Therefore, the PCD tools and tools with diamond coatings are advisable for machining of aluminum alloys, in particular hard-particles alloys. New kinds of cutting inserts, based on material composites, should minimize abrasive wear, built-up-edge, forces and also keep the acceptable surface quality and enhance tool life [62,63].

The PCD and sintered carbide cutting tools are commonly applied during the hard-machining process [64,65], because of a high hardness level [66,67]. The primary disadvantage of PCD material is its relatively low maximum cutting temperature of 600 °C and the affinity to ferrous materials [68]. Unfortunately, the high cost of diamond sintering causes that PCD tools are more expensive than others; moreover, fracture toughness is unsatisfactory compared to other materials [69]. Besides, PCD cutting tools are characterized by difficulties in obtaining similar geometries of the surface during the manufacturing process [70]. What is also essential, the high brittleness of PCD can cause to the premature tool catastrophic failure [71,72]. Regardless of the presented drawbacks, PCD cutting tools are widely used because of very high abrasion resistance [73,74]. In case of Al 4xxx alloys with a high Si content and high chemical reactivity, the wear intensity increases, which demands the usage of PCD [75]. The next very popular tool material is tungsten carbide (WC). These materials have high hardness, excellent abrasion resistance and high crack resistance. By using PCD particles, it is possible to increase the hardness and the material fracture toughness [76].

Some latest researches focus on the production of dual cutting tool materials with the benefits of tungsten carbides and hard PCDs. The WCCo/PCD composite (DDCC—diamond dispersed cemented carbide) is an example of a modern tool material. The DDCC is a composite containing the low volume of PCD (polycrystalline diamond) in the WCCo matrix [69]. In the production process of DDCC, the PCD is provided as the MBD4 powder with a nano or micrometric grain size [69]. The hot isostatic pressing sintering method is applied as the production method [77]. Compared to other methods, the primary attribute of pulse plasma sintering is the high energy provided in a short time [78,79]. Conventional sintering conducted in the range of temperatures between 1400 and 1500 °C could result in the unstable diamond phase and graphitization [80].

Apart from limited researches focused on the production of the DDCC materials, their cutting performance is still not investigated [81]. Thus this work presents the identification of some fundamental cutting indicators, as tool wear mechanisms and surface roughness formation in the transverse turning of Al-Si alloy. The carried out experiments included the inspections of machined surface topographies after grooving with different cutting parameters. In addition, the tool life and wear tests of DDCC inserts were conducted during grooving process and the obtained results were compiled with values reached during machining with cemented carbide tools. The results can be selected for an effective selection of machining parameters during cutting of Al-Si alloys with DDCC materials.

## 2. Experimental Details

### 2.1. Research Plan

**Figure 1:**
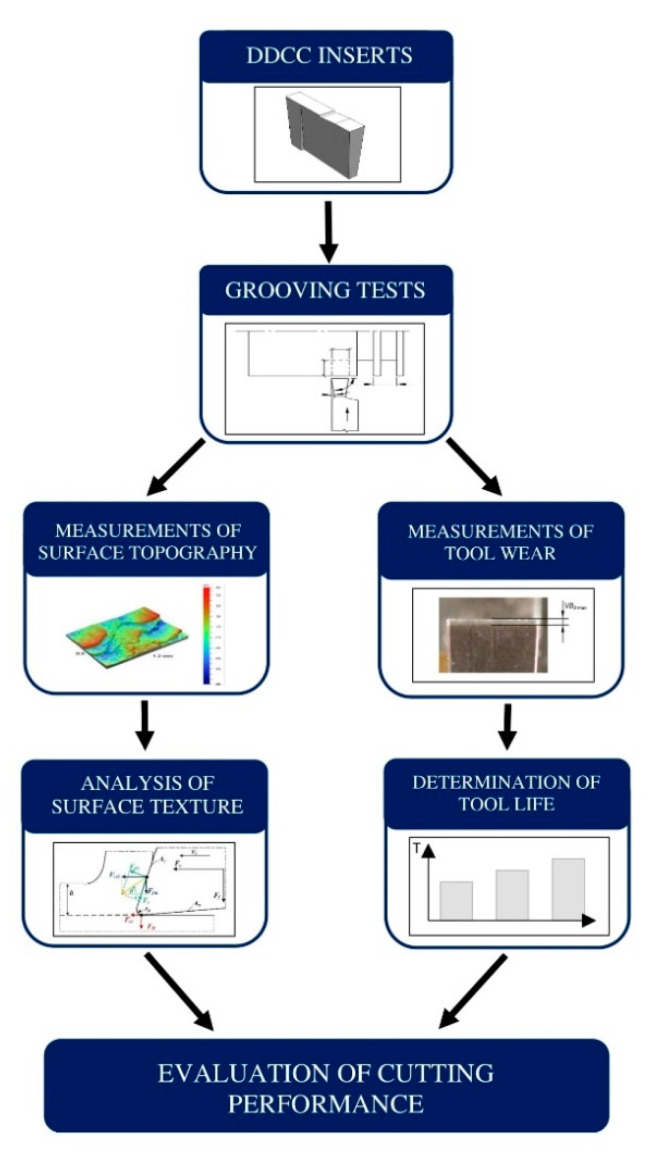
The flowchart of a carried out experiment.

The basic technological effects of a precise grooving of AlSi13MgCuNi alloy with a novel uncoated WCCo/PCD (DDCC) inserts were evaluated as part of this study. Measurements of tool wear for a various cutting parameters during the external grooving process and evaluation of surface texture were performed during the experiments. Figure 1 presents the flowchart of the carried out experiment.

The grooving process with an orthogonal kinematics of Al-Si alloys has been selected, since it is very often employed during manufacturing of grooves in pistons intended for exhaustive engines. The aluminum alloys containing silicon are among the most popular construction materials applied in many industrial applications due to relatively low mass and high strength, excellent thermal and electrical conductivities [46]. Moreover, its corrosion-resistant properties and tendency to passivate increase its use in welded tanks, fuel and oil supply lines and propellers [46,47]. In particular, the aluminum alloys containing Si are categorized as difficult-to-cut materials. Therefore, the orthogonal non-free grooving was employed due to generation of high cutting loads. Therefore, this research can be applied for the experimental verification of DDCC tools during machining tests dynamic conditions.

### 2.2. Production of DDCC Composite

The WCCo/PCD composite (DDCC) used in this study was produced with a use of PPS technique. The WC and Co powders with an addition of MBD4 diamond powder were applied in the manufacturing process (see Table 1). The mixing process of powders was conducted in two steps—preparation of the WC6Co and combination WC6Co with diamond particles. Sintering was made in a PPS apparatus in the pressure of 5 × 10^−3^ Pa [82]. Compared to other methods, the PPS enables the obtainment of high energy equal to 600 MW, provided in a short time. Sintering process in the conventional conditions, conducted in the range of temperatures between 1400–1500 °C could result in the unstable diamond phase and graphitization [80].

The uncoated DDCC composite samples were ground with PCD grains (with size of 20–30 µm, Warsaw University of Technology, Warszawa, Poland) with a ceramic adhesive Ba23 (Warsaw University of Technology, Warszawa, Poland). As a consequence, the surface roughness was influentially reduced (*Ra* = 1.28–1.4 µm, *Rz* = 7.05–7.9 µm—before grinding; *Ra* = 0.018–0.04 µm, *Rz* = 0.126–0.150 µm—after grinding) [83]. The medium WC grain size in the DDCC composite with a 6 wt.% of Co is 0.42 μm [82]. According to Rosinski et al. [82], the hardness of the DDCC is 23 GPa (approx. 2345 HV). This value is significantly higher than values reached for the nano-cemented carbides [80]. In addition, its fracture toughness is approx. equal to 9 MPa∙m^1/2^ (Table 1.), thus it is 50% higher than values reached for the polycrystalline diamonds.

**Figure 2:**
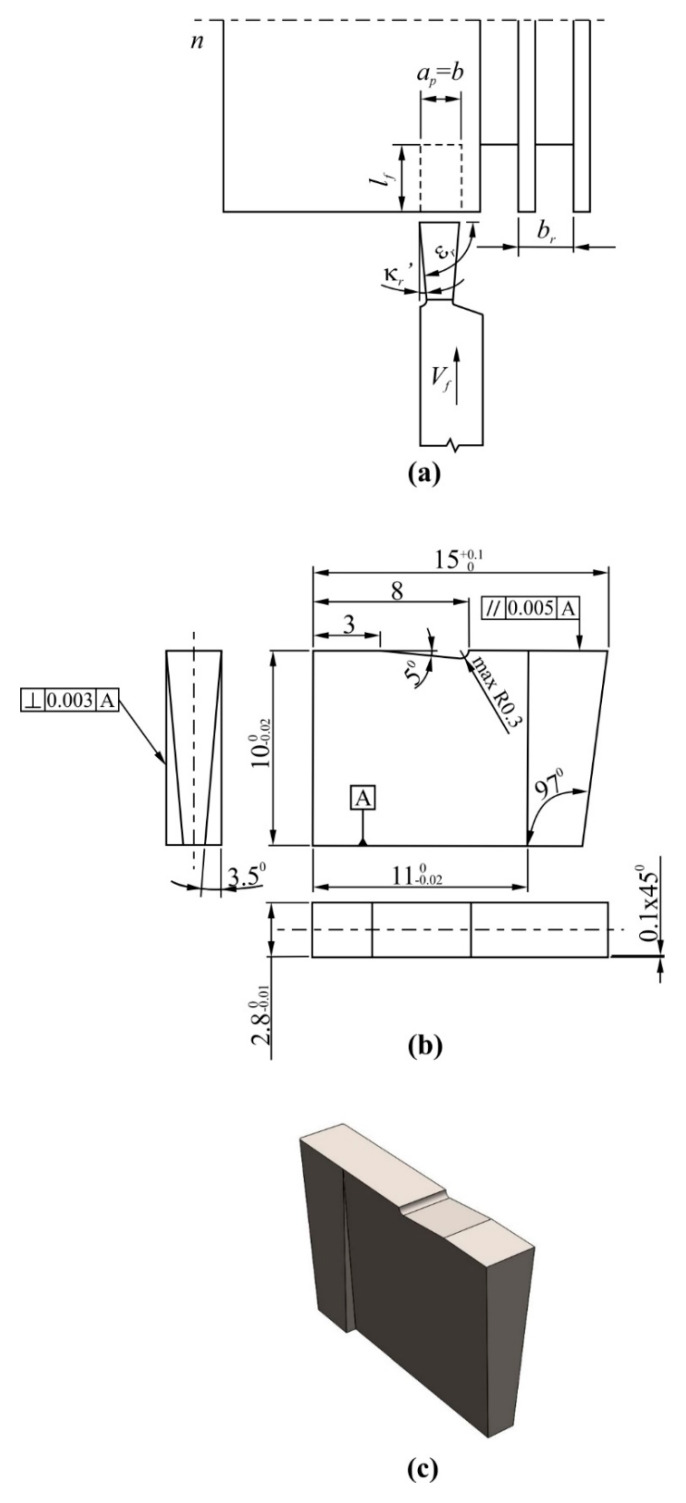
The grooving with DDCC inserts: (**a**) grooving kinematics, (**b**) geometry of tool (values given in millimeters) (**c**) cutting insert in isometric presentation.

Figure 2 shows the cutting insert geometry applied during grooving process of aluminum-silicon alloy.

### 2.3. Turning Tests

Samples made of AlSi20MgCuNi alloy, containing (in wt.%): 20% Si, 1.3% Cu, 1% Ni, 0.8% Mg, 0.2% Mn and rest Al, were used during the grooving tests. The cylindrical workpiece samples had diameter of *d* = 130 mm. The workpiece material was characterized by the following mechanical properties—tensile strength 320 MPa, shear strength 260 MPa, hardness 137 HV and elongation 6.7%. The precise CTX 310 Ecoline 560E lathe (DMG, Pleszew, Poland) was employed during cutting trials. A semi-synthetic coolant concentrate (Statoil ToolWay ST) with 6% content, density of 990 kg/m^3^ and a working pH of 9 was used during tests. The cutting path in feed direction was *l_f_* ≈ 3 mm and the pick feed was *b_r_* = 1.9 mm. The grooving parameters applied during the tests are shown in Table 2 and Table 3. Diagram of the grooving process is shown in Figure 2a. The uncoated H3 and H10 inserts were also employed in tests, since they are very often employed for the machining of Al-Si alloys The main difference between the H3 and H10 cemented carbides involves the level of hardness and bending strength. The selected properties of a H3 and H10 sintered carbides are shown in Table 4.

The geometry of all tested cutting tool was as follows—main cutting edge angle *κ_r_* = 90°, corner radius *r_ε_* = 0.1 mm (chamfer 0.1 mm × 45°), orthogonal rake angle *γ_o_* = 1°, orthogonal flank angle *α_o_* = 9°, length of main cutting edge *l* = 2.3 mm, angle of inclination of the main cutting edge *λ_s_* = 0.

### 2.4. Inspection of Tool Wear and Calculation of Tool Life

**Figure 3:**
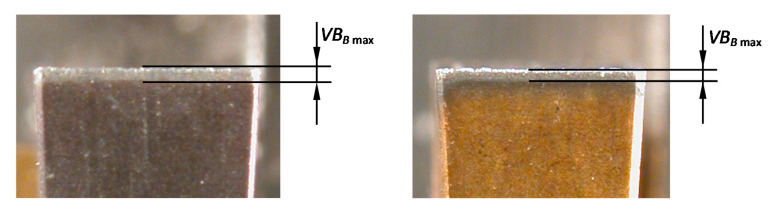
Tool flank wear measurement for different inserts.

The stereoscopic and scanning electron microscopes (SEM, Tescan Vega, Brno, Czech Republic) were applied for the visual inspections of tools after grooving. The stereoscopic microscope was employed to the evaluation of maximal flank wear width located on the straight section of the leading cutting edge *VB_Bmax_* (see Figure 3). The scanning microscope was employed for the inspection of a BUE onto the tool flank and rake faces, as well as the cutting edge. Moreover, the identification of a crater wear on the rake face was made with the use of SEM. The length of crater wear was measured perpendicularly to the length of a cutting edge.

The time of machining for the number of tool passes equal to *p* was expressed by the relation:

(1) $$t_{s}=\frac{l_{f}}{fn}p.$$

The *l_f_* is a path of machining in the direction of feedrate [mm], *n* is a spindle rotation speed [rpm].

The path of machining in the main (circumferential) direction, considering number of tool passes equal to *p* can be determined by equation:

(2) $$L_{c}=\sum_{j=1}^{p}l_{cj}.$$

The symbol *l_c_* in Equation (2) denotes the cutting path length in the main direction for a one pass. The following equation determines the value of the *l_c_* factor:

(3) $$l_{c}=\pi \sum_{i=1}^{\frac{l_{f}}{f}}d-2\left(i-1\right)f.$$

The critical wear of *VB_cr_* = 0.1 mm was adapted to calculate the tool life. The value of the selected *VB_cr_* was employed from the recommendations used in automotive industry to the finishing of grooves intended for a piston rings. Moreover, Ozel et al. [84] stated that during longitudinal finishing turning of metals, the dullness criterion in relation to the flank wear can be selected as 0.1 mm.

In the carried out experiments, one tool wear trial per the tested insert and selected input parameters combination was employed. It should be noted that the tool life for a given tool grade and the same workpiece/input parameters combination can be variable in the range of different inserts. This is usually attributed to some alterations in cutting insert geometry/properties, changes in workpiece surface finish and properties, as well as some random factors occurring during cutting. Nevertheless, according to Oraby and Alaskary [85], during turning of metals, in the range of critical flank wear equal to 0.2 mm, the variability of tool wear per consecutive inserts is not exceeding the 15%. Therefore, the influence of tool wear variability induced by the application of distinct cutting inserts was neglected in the current study.

### 2.5. Evaluation of the Machined Surface Topography

The surface roughness and topography measurements were conducted using the stylus (Hommel-Etamic T8000, Hommel Etamic, Jena, Germany) and the optical (Veeco NT 1100, Veeco, Plainview, NY, USA) profile meters. Measurements were repeated five times on a different areas of the tested surfaces. The following settings have been applied during roughness measurements—elementary segment length *l_r_* = 2.5 mm, measuring segment length *l_n_* = 10.0 mm, filter cut-off wavelength *λ*c (cut-off) = 2.5 mm. Based on obtained profiles, the EVOVIS software (Version 1.38, Hommel Etamic, Jena, Germany) has been applied to calculate the spatial surface roughness parameters. Optical measurements were carried out with a 5.1-fold magnification. The area of the scanned surface was 0.9 mm × 1.2 mm, while the distance of vertical points of 1.65 µm.

## 3. Results and Discussion

### 3.1. Evaluation of Tool Wear and Tool Life

**Figure 4:**
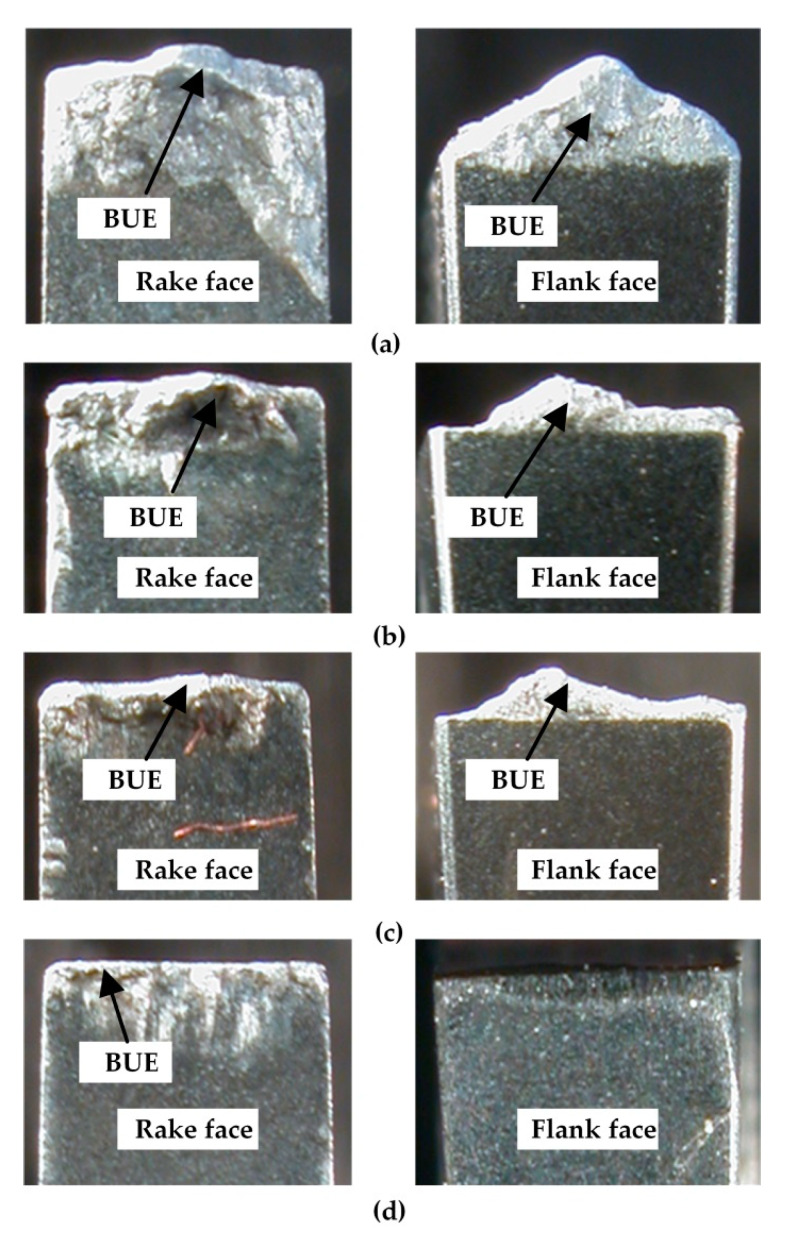
Microscopic images of the DDCC inserts after cutting with various input parameters: (**a**) *v_c_* = 180 m/min, *f* = 0.2 mm/rev, *VB_B_* = 0.04 mm; (**b**) *v_c_* = 400 m/min, *f* = 0.2 mm/rev, *VB_B_* = 0.04 mm; (**c**) *v_c_* = 600 m/min, *f* = 0.2 mm/rev, *VB_B_* = 0.04 mm; (**d**) *v_c_* = 800 m/min, *f* = 0.15 mm/rev, *VB_B_* = 0.05 mm.

In the first stage, the machining performance of DDCC inserts was analyzed in terms of tool wear and tool life. The microscopic images of cutting edges (Figure 4) show the appearance of built-up-edge (BUE), independently on selected cutting speed during cutting. According to Barzani et al. [31], the appearance of BUE during turning of Al-Si alloy is strictly attributed to its ductility; moreover, the decline in Si content leads to a higher adhesion. Primarily, the intensification of adhesion phenomenon is induced by the appearance of normal and tangential stresses caused by cutting forces. Moreover, the high friction and load concentration in a small contact area between tool and workpiece and almost "chemically pure" newly created chip surface, as well as relatively active rake face are also the sources of adhesion intensity.

It was shown that cutting speed significantly affects the adhesion intensity on tool working part. The appearance of this phenomenon is manifested by an intense generation of a double built-up-edge simultaneously on flank and rake faces. The growth of *v_c_* factor leads to the decline in BUE intensity. This observation is attributed to relations between the cutting speed and the cutting temperature, which in turn significantly affects the adhesion intensity. According to Józwik and Domińczuk [86], an intensification of BUE phenomenon has a place within a certain range of cutting speeds, feeds and corresponding temperatures, as well as the cutting forces. The most intensive growth corresponds to the cutting speed at which the temperature in chip-tool interface is approx. 300 °C. However, the total disappearance of BUE is observed at cutting speed, at which the contact temperature is around 600 °C. On the other hand, the decrease in BUE intensity can be also attributed to the decline in the friction coefficient between the tool and workpiece, together with a growth of cutting speed, as reported in Reference [87].

Figure 4a–c show that in case of cutting speeds *v_c_* in a range between the 180 m/min and 600 m/min, the height of BUE can exceed even 1 mm. However, during grooving with a cutting speeds equal to at least 800 m/min, the slight BUE was formed mainly on the tool rake face (Figure 4d). Thus, the further wear tests were employed in a range of cutting speeds: *v_c_* ≥ 800 m/min.

**Figure 5:**
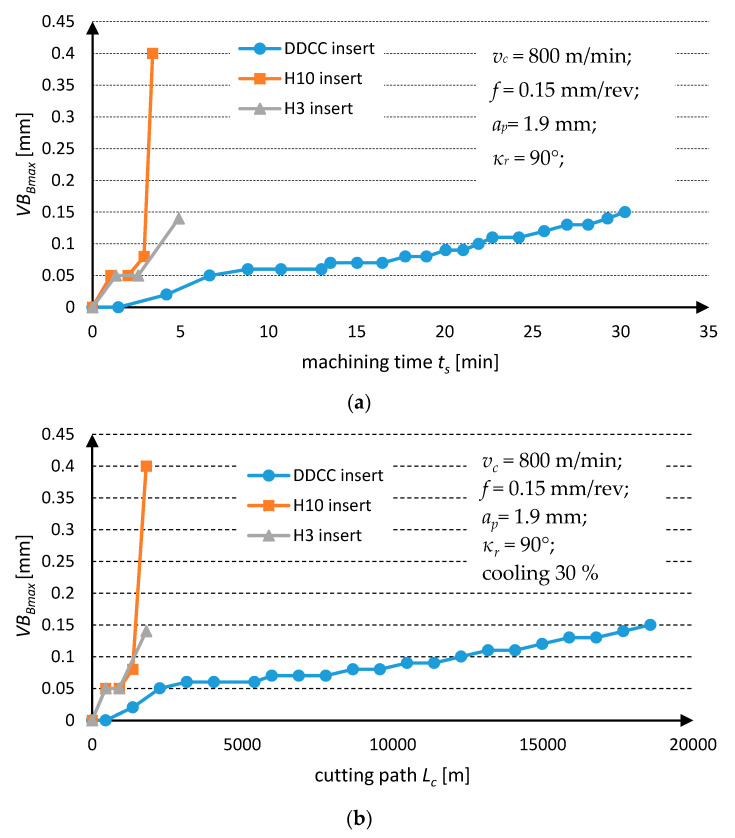
Tool wear curves for DDCC, H3 and H10 inserts obtained during grooving with *v_c_* = 800 m/min and *f* = 0.15 mm/rev in function of (**a**) cutting time; (**b**) cutting path.

The Lorenz wear curves, depicted in Figure 5 show that during grooving of Al-Si alloy with DDCC insert, in the range of *v_c_* = 800 m/min and *f* = 0.15 mm/rev, the almost linear growth of a flank wear in function of cutting time *t_s_* and path *L_c_* is found. This observation indicates the occurrence of a typical continuous and moderate wear growth, characteristic for an appearance of abrasion and adhesion wear mechanisms [88,89].

Nevertheless, in the case of cutting with H3 and H10 cemented carbides, the rapid and stepped growth of tool wear has been observed, which resulted in occurrence of catastrophic tool failure after cutting time less than 5 min. It reveals an occurrence of an excessive cutting load, due to interaction of hard Si particles contained in Al-Si alloy with the cutting tool during cutting, which consequently can cause the growth of stresses above the local strength limit of a material.

**Figure 6:**
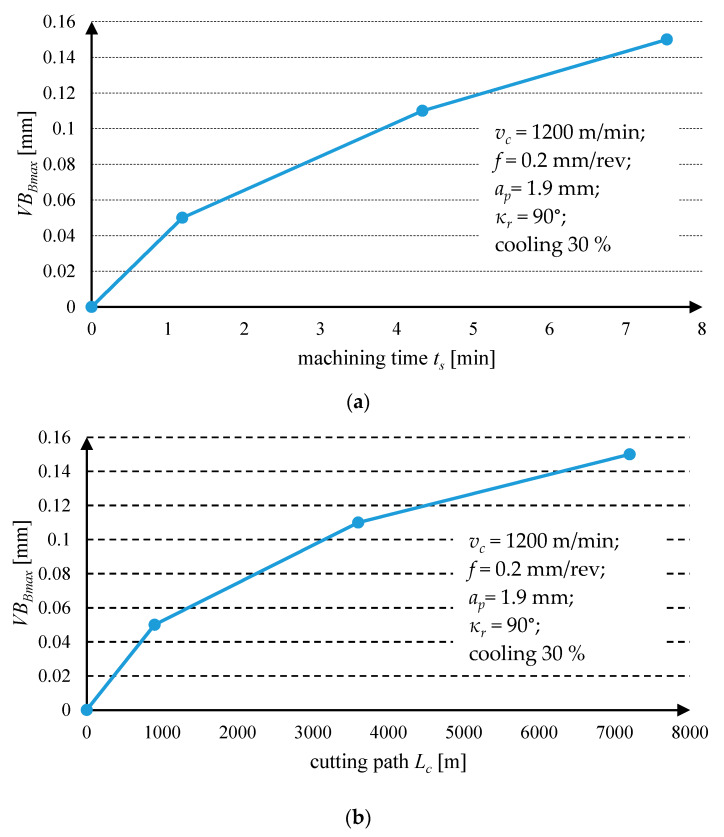
Tool wear curves for DDCC insert obtained during grooving with *v_c_* = 1200 m/min and *f* = 0.2 mm/rev in function of (**a**) cutting time; (**b**) cutting path.

The wear test of DDCC inserts has been also conducted during grooving with higher cutting speed *v_c_* = 1200 m/min and feed *f* = 0.2 mm/rev (Figure 6). In this case, a significantly higher wear intensity has been found, comparing to that observed during cutting with *v_c_* = 800 m/min and *f* = 0.15 mm/rev. The observed wear curve had a non-linear/exponential progress with cutting time and path, suggesting the appearance of an accelerating wear (failure wear region). This observation can suggest that the growth of a cutting temperature (resulting from a growth of cutting speed), can induce an intense wear mechanisms (e.g., abrasion or diffusion) of DDCC tools.

**Figure 7:**
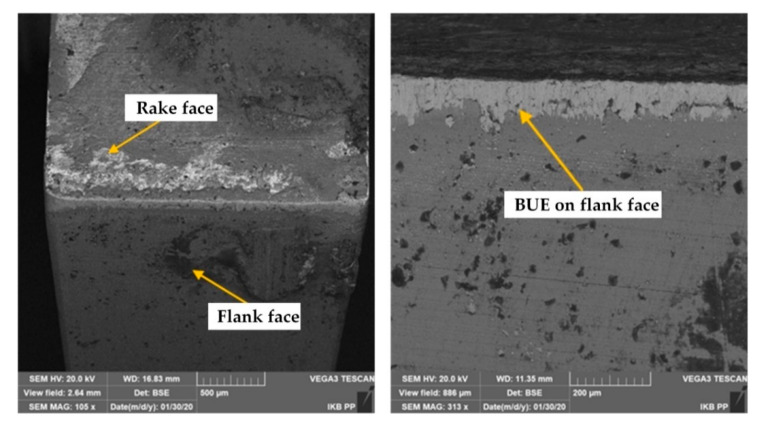
The scanning electron microscope (SEM) images of DDCC inserts after grooving with *v_c_* = 800 m/min, *f* = 0.15 mm/rev, *t_s_* ≤ 30 min.

**Figure 8:**
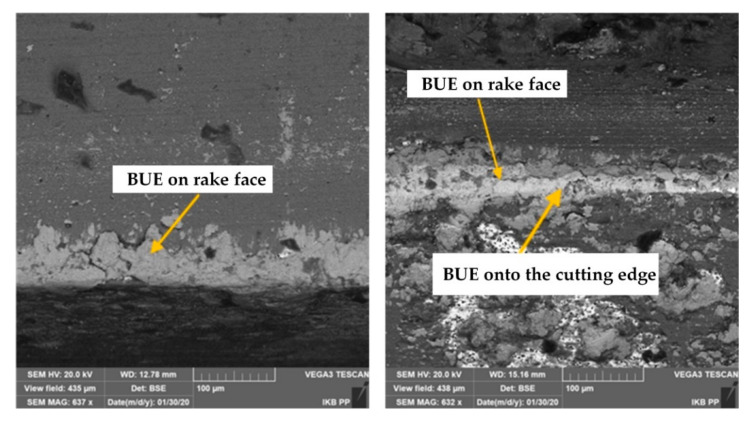
The SEM images of DDCC inserts after grooving with *v_c_* = 1200 m/min, *f* = 0.2 mm/rev, *t_s_* ≤ 2 min.

**Figure 9:**
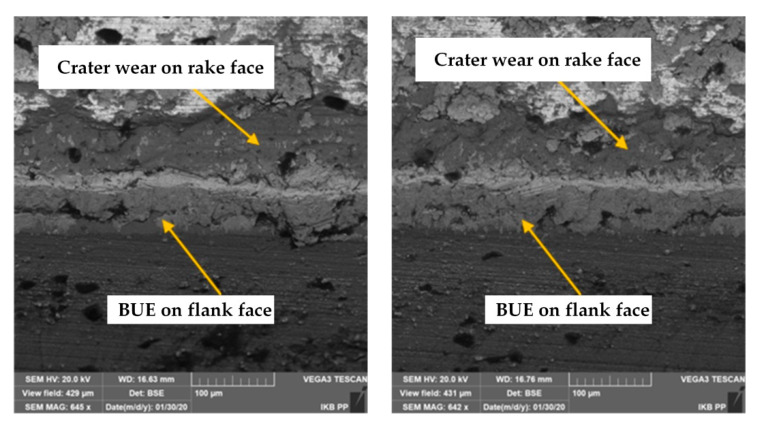
The SEM images of DDCC inserts after grooving with *v_c_* = 1200 m/min, *f* = 0.2 mm/rev, *t_s_* ≤ 8 min.

The thorough characterization of tool wear mechanisms during grooving with DDCC inserts was conducted with the SEM images of cutting edges (Figure 7, Figure 8 and Figure 9), obtained after the machining process. During grooving with *v_c_* = 800 m/min, *f* = 0.15 mm/rev and in a range of cutting time *t_s_* ≤ 30 min, the stable BUE was located onto the cutting edge with the maximal width not exceeding 0.1 mm (Figure 7). The appearance of relatively low BUE widths reveals the appearance of a stable adhesive wear mechanism, which contributes to the moderate tool wear growth in a function of cutting time. In case of cutting with *v_c_* = 1200 m/min and *t_s_* < 2 min, the stable BUE on the rake and flank faces, as well as along the cutting edge was also found (Figure 8). Nevertheless, in a range of cutting time *t_s_* < 8 min, apart from intense built-up-edge, the crater wear on the tool rake face has been observed (Figure 9). In some cases the width of crater was higher than 0.1 mm. Its appearance contributes significantly to the intense wear rate of DDCC inserts (see Figure 6).

The crater on the rake face is caused by a cyclic formation and destruction of an adhesive joints. This periodic phenomenon can consequently lead to exceeding the local tool material strength limit and thus the material loss in a form of craters. In addition, the flowing of very hard Si particles along the rake face during the chip flow causes tool material chunking and thus the crater wear. This phenomenon can be additionally supported by the material diffusion, which is initiated by the chemical purity of tool-chip contact zone and high cutting temperatures (>800 °C), induced by a cutting process in a range of high cutting speeds.

The basic mechanisms of tool wear during cutting of high-silicon Al-Si alloys are—abrasion as a result of scratching the cutting edge with a separated hard silicon particles, as well as microchipping of tool working part, associated with periodic removal of BUE from the tool [32]. In addition, an intense formation of BUE during machining of Al-Si alloys can be induced by an intense flank wear and cutting temperatures exceeding 500 °C [33].

**Figure 10:**
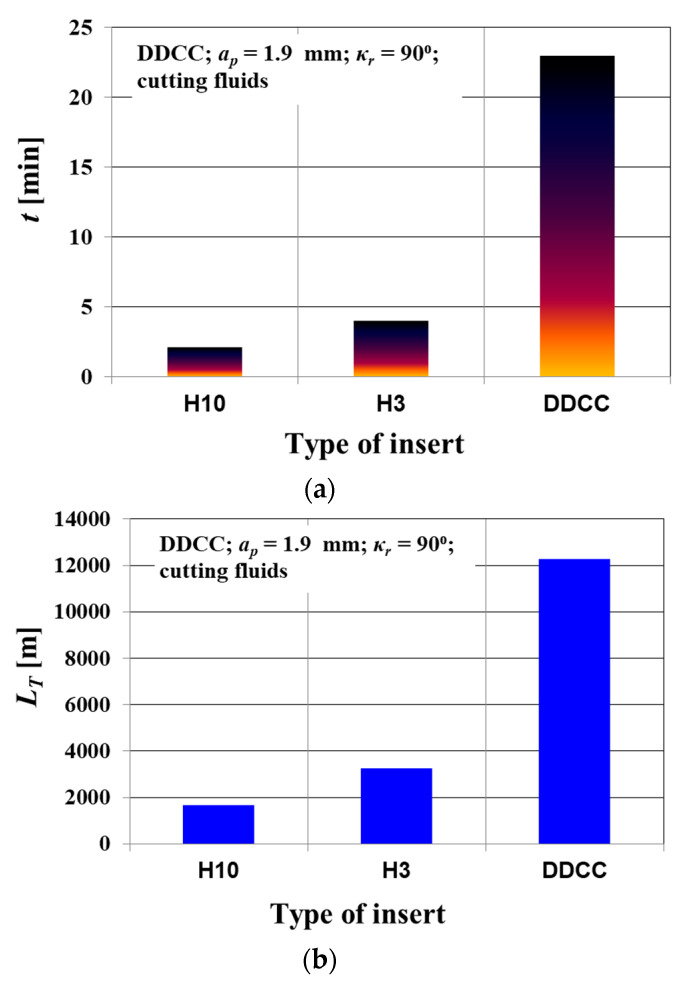
Durability of tested cutting inserts within the range of *v_c_* = 800 m/min, *f* = 0.15 mm/rev: (**a**) comparison of the tool life of DDCC, H3 and H10 cemented carbides; (**b**) cutting path over the tool life for DDCC, H3 and H10 cemented carbides.

Figure 10 shows the comparison of tool life *t* and the cutting path in a range of tool life *L_T_*, obtained after the turning tests with DDCC and cemented carbide (H3, H10) inserts. It was observed that in the case of DDCC inserts, the tool life and cutting path in a range of tool life were respectively 23 min and 12,279 m.

Considering the cemented carbide tools, the following values were achieved—*t* = 5 min and *L_T_* = 2500 m for H3 cemented carbide and *t* = 4 min and *L_T_* = 4500 m for H10 cemented carbide. This means that the use of DDCC inserts during grooving of Al-Si allows obtaining more than 5 times longer tool life and almost 3-fold increase of cutting path compared to values obtained during turning of H3 and H10 cemented carbide tools.

**Figure 11:**
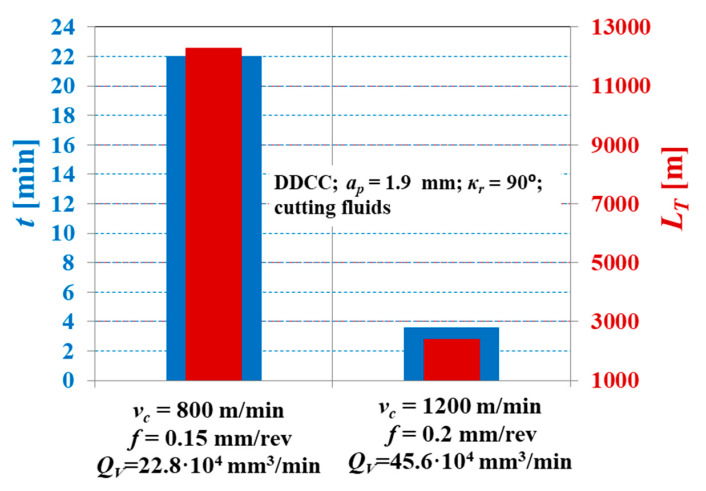
The comparison of tool life and cutting path over the tool life for DDCC inserts after grooving with various cutting parameters.

Figure 11 depicts the tool life *t* and the cutting path in a range of tool life *L_T_* for DDCC composite inserts after grooving with different cutting parameters. It was observed that an increase in the *f* value from 0.15 to 0.2 mm/rev and *v_c_* from 800 to 1200 m/min results in a 2-fold increase in material removal rate *Q_V_*.

On the other hand, the use of increased cutting parameters (*f* = 0.2 mm/rev; *v_c_* = 1200 m/min) causes a 6-fold shortening of tool life and a 5-fold decrease in the cutting path over the tool life, compared to the values obtained during turning with *f* = 0.2 mm/rev; *v_c_* = 1200 m/min. This indicates that the moderate growth of a grooving productivity with DDCC inserts can lead to the intense decline in tool life. Therefore, this aspect should be considered during the selection of an optimal cutting parameters for a DDCC tools.

**Figure 12:**
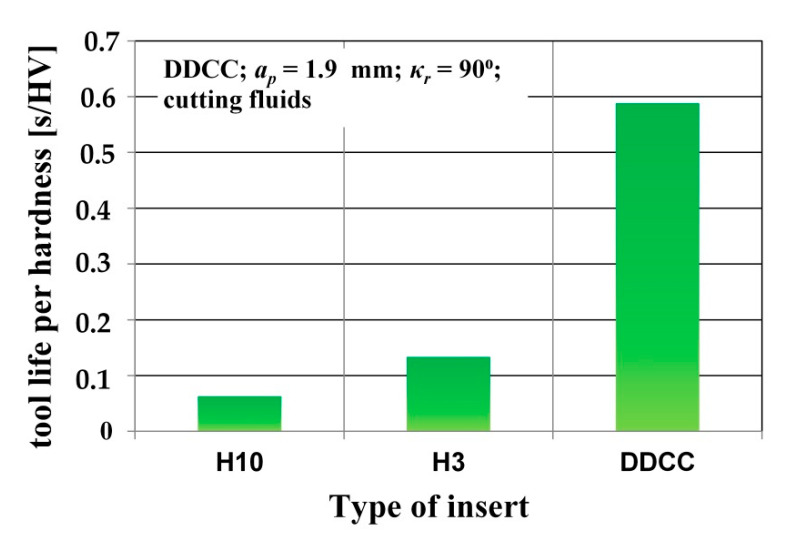
Tool life per hardness for tested cutting inserts within the range of *v_c_* = 800 m/min, *f* = 0.15 mm/rev.

In order to define the relations between the tool life and its mechanical properties (mainly hardness), the charts depicting tool life per tool material hardness were developed for the tested inserts (Figure 12). These charts characterize the intensity of tool material hardness effect on the obtained tool life. During cutting of a particular material, tool life is considerably affected by tool hardness. However, during machining processes also other tool properties, as temperature resistance, fracture toughness and bending stress have important effect on the tool durability. In case when the tool life per hardness ratio values for tools with distinct hardness will be at the same level, the tool life could be not affected by abrasion wear mechanisms (correlated directly with tool hardness) but phenomena (e.g., intense adhesion, dynamic loads, high temperature, etc.) correlated with other tool properties. In the carried out studies, the highest difference in hardness level of tool materials was of 30%. Nevertheless, the largest difference in tool life per tool material hardness ratio was equal to 930%. This vast difference denotes that cutting ability of tested tool materials during grooving of Al-Si alloy is only marginally affected by tool material hardness. Therefore, the possible reasons of differences in tested materials durability during machining of AlSi20MgCuNi alloy can be attributed to other tool material properties, as strength, fracture toughness and maximal working temperature. However, the verification of this observation requires further studies on tool material properties.

### 3.2. Evaluation of Surface Topography

**Figure 13:**
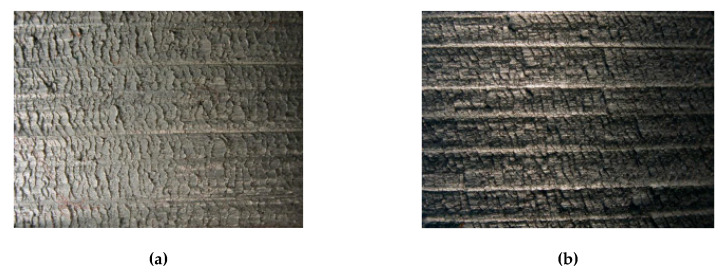

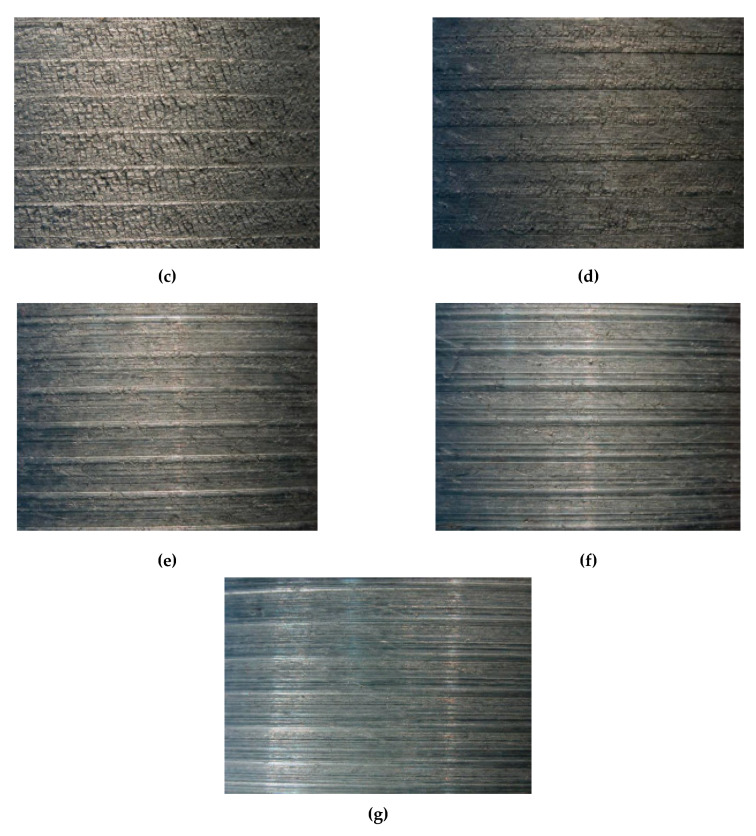
Images with a 4-fold magnification of a surfaces after grooving with DDCC insert in the range of: (**a**) *v_c_* = 50 m/min; (**b**) *v_c_* = 100 m/min; (**c**) *v_c_* = 200 m/min; (**d**) *v_c_* = 400 m/min; (**e**) *v_c_* = 600 m/min; (**f**) *v_c_* = 800 m/min; (**g**) *v_c_* = 1000 m/min.

The machined surface topography constitutes the fundamental effect of the machining process and simultaneously one of primary cutting tool efficiency indicators. Thus, in this section the machined surface microscopic images (see Figure 13), together with 3D surface topographies and 2D surface roughness profiles obtained after grooving with various cutting parameters are characterized.

Figure 13 depicts an image of a machined surface after the grooving of Al-Si alloy with a DDCC insert for a various cutting speed values. The formation of a surface texture during external grooving process is not affected by a value of feed, since the feed marks formed during the considered position of a cutting edge are being completely removed during the subsequent workpiece revolution. Therefore, the formation of a surface irregularities during grooving process can be affected mainly by a micro-profile of a cutting edge, machining system vibrations, material decohesion mechanisms correlated with interactions between tool and workpiece or the discontinuities/phase composition of a workpiece.

**Figure 14:**
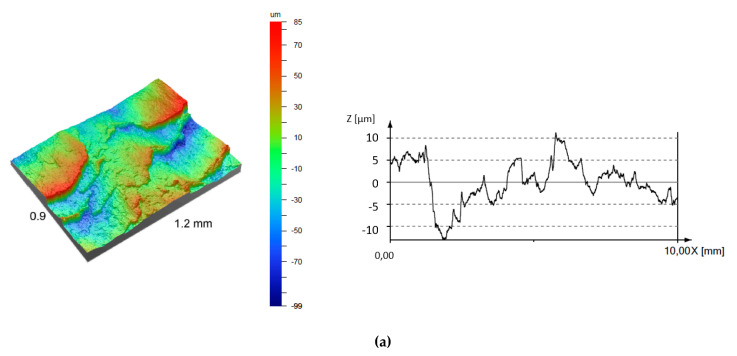

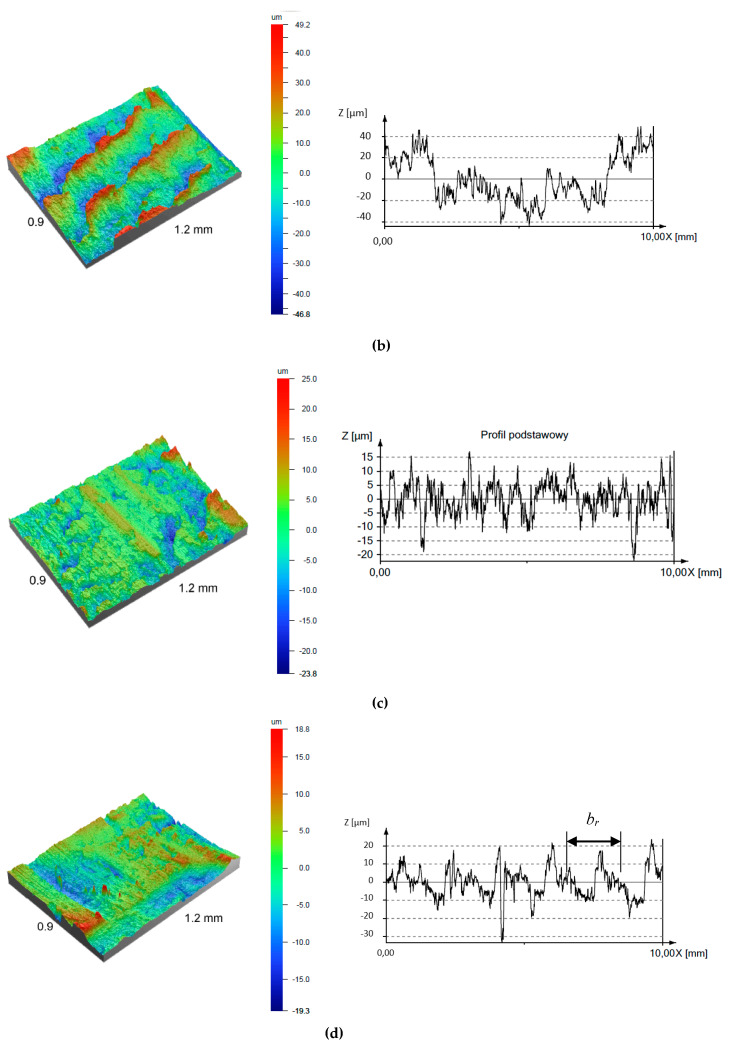

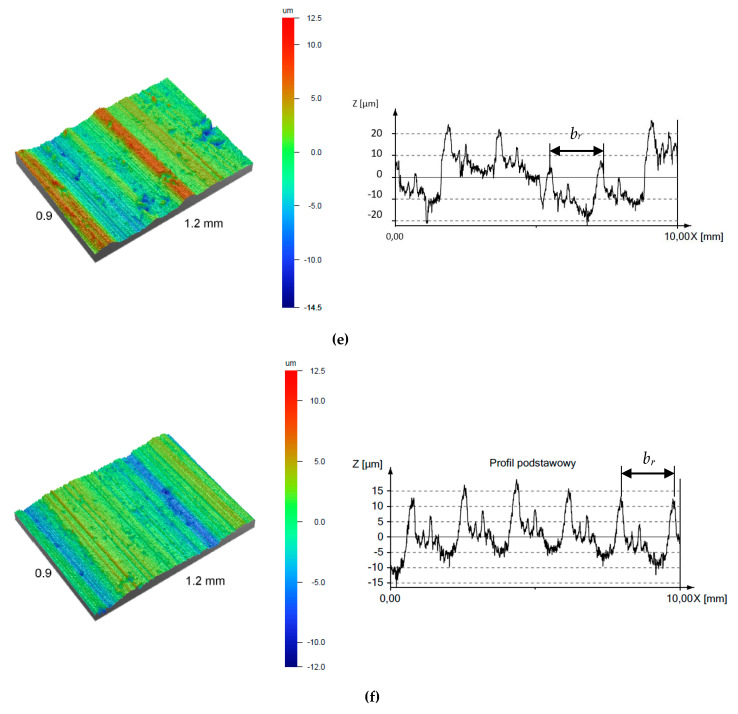
3D topographies and 2D surface profiles of a machined surfaces of the AlSi13MgCuNi alloy after grooving with a DDCC insert in the range of: (**a**) *v_c_* = 50 m/min; (**b**) *v_c_* = 100 m/min; (**c**) *v_c_* = 200 m/min; (**d**) *v_c_* = 400 m/min; (**e**) *v_c_* = 600 m/min; (**f**) *v_c_* = 800 m/min.

**Figure 15:**
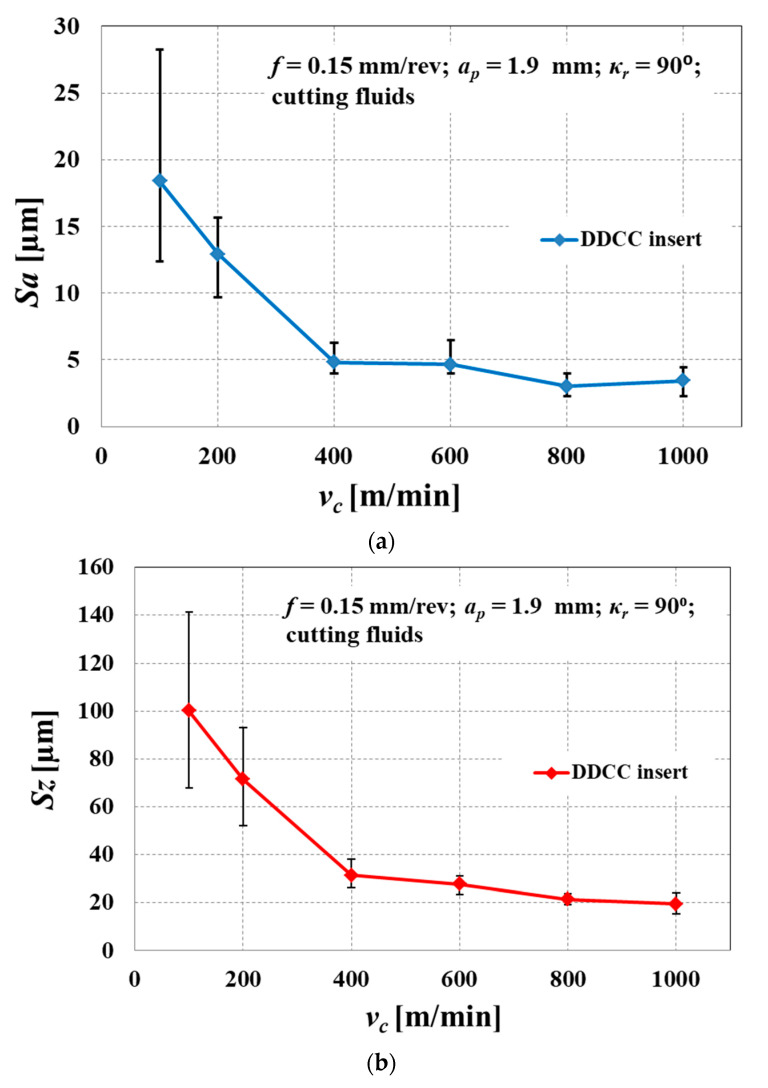
The influence of cutting speed on surface roughness of AlSi13MgCuNi alloy during grooving with a DDCC insert: (**a**) *Sa* parameter; (**b**) *Sz* parameter.

It was shown that machined surfaces formed in a cutting speed range of 50 m/min ≤ *v_c_* ≤ 400 m /min are irregular and characterized by an intensive cracks and exfoliations. These observations can be also confirmed by a 3D surface topographies, surface profiles (Figure 14) and determined surface roughness parameters (Figure 15). Nevertheless, for a higher cutting speeds (*v_c_* > 400 m/min), the machined surface becomes smoother and the number of cracks and exfoliations is being significantly reduced (see Figure 13e–g and Figure 14d–f).

It should be noted that the cutting speed range characterizing an appearance of intense surface fractures overlaps with a cutting speed range of intense BUE formation on the cutting edge. Thus, the surface quality is significantly affected by an adhesion phenomenon, which causes the periodic formation of an adhesive joints of the workpiece on the cutting edge and subsequently the destruction of these joints and re-deposition on the surface after cutting. In addition, the appearance of cracks on the surfaces machined with a lower cutting speeds can be correlated with a high tensile residual stresses induced by a values of cutting forces.

According to Akyuz [90], the growth of cutting speed leads to the decline in cutting forces during turning of Al-Si alloys with the polycrystalline diamond (PCD) inserts. Therefore, in a range of lower cutting speeds the higher values of cutting forces can lead to the higher residual stresses and thus to the appearance of cracks on the surface after machining.

Ultimately, the Si content in the tested alloy can cause the silicon grain chunking during machining, which can consequently lead to the deterioration of a surface finish.

The analysis of surface topographies formed during grooving with higher cutting speeds (*v_c_* > 400 m/min, Figure 14d–f) reveals an occurrence of a periodic dominant irregularity peaks with the wavelength equal to approx. 1.9 mm. The value of this distance corresponds directly to the employed pick feed *b_r_* value during the grooving tests. Therefore, the source of these irregularities is the side plastic flow of a workpiece, which is induced by material elastic/plastic deformations and ploughing mechanisms. Nevertheless, during the analysis of a singular groove turning process, no pick-feed is applied and thus the formation of a surface profile on the circumference of workpiece will not be affected by a material plastic side flow. In case of a lower cutting speeds (Figure 14a–c), no effect of side plastic flow is visible, since the formation of surface irregularities is dominated by an intense adhesion and formation of cracks.

Figure 15 shows the surface roughness spatial parameters (*Sa*, *Sz*) in function of *v_c_*. The observed non-linear decline in *Sa* and *Sz* parameters values together with a growth of *v_c_* is consistent with a literature reports [90,91]. It can be also seen that error bars, describing the range of a measured *Sa* and *Sz* values reach influentially higher values in a range of a lower cutting speeds, (50 m/min ≤ *v_c_* ≤ 200 m/min). This observation reveals that the appearance of an intense BUE and formation of cracks on the surface can cause the random distribution of a surface roughness in various areas of a machined surface.

Ultimately, the application of a novel DDCC inserts enables the obtainment of a minimal surface roughness parameters *Sa* = 3.4 μm and *Sz* = 19.4 μm during grooving of Al-Si alloy with the cutting speed of *v_c_* = 1000 m/min and *f* = 0.15 mm/rev. These values are comparable to those obtained during longitudinal turning of Al-Si alloy with cemented carbide TiN coated inserts [31]. Thus, in terms of a surface finish, the novel DDCC cutting tools can be employed as an alternative to the conventional coated cemented carbide tools.

## 4. Conclusions

In this paper the cutting performance experiments of the modern WCCo/PCD (DDCC) tools during the machining of Al-Si alloy were presented. The carried out tests included the inspections of the tool wear and assessment of tool life. Moreover, the research included the evaluation of the machined surface topography. On the basis of the research, the following conclusions were given.

(1) The adhesive DDCC tool wear manifested as the BUE was the dominant wear mechanism in the range of investigated input parameters. However, the intensity of this phenomenon was significantly reduced with an increase in cutting speeds. During grooving with *v_c_* = 800 m/min and *f* = 0.15 mm/rev, the stable BUE was located onto the cutting edge with the maximal width not exceeding 0.1 mm, which contributes to the moderate tool wear growth in a function of cutting time. However, during grooving with a cutting speed of *v_c_* = 1200 m/min, the crater wear on the tool rake face has been also observed, whose appearance contributed influentially to the intense wear rate of DDCC inserts.
(2) The selection of *v_c_* = 800 m/min and *f* = 0.15 mm/rev during grooving of AlSi13MgCuNi alloy with DDCC inserts enables 5 times longer tool life and almost 3-fold increase of cutting path compared to values obtained during grooving with H3 and H10 cemented carbide inserts. This observation proves the significantly higher cutting performance of inserts made of DDCC during machining of AlSi13MgCuNi alloy compared to that reached for a cemented carbides (H3, H10).
(3) It has been found that increase of cutting speed contributed to the influential improvements of surface quality after grooving with the DDCC inserts. During machining in a cutting speed range of 50 m/min ≤ *v_c_* ≤ 400 m/min, machined surfaces were irregular and characterized by an intensive cracks and exfoliations. On the other hand, during machining with higher cutting speeds (*v_c_* > 400 m/min), the machined surface became smoother and the number of cracks and exfoliations was significantly reduced. This observation reveals the significant role of BUE formation during grooving with DDCC inserts.
(4) Characterization of tool wear and tool life revealed that the feed value of *f* = 0.15 mm/rev and cutting speed in a range of 800 m/min ≤ *v_c_* ≤ 1000 m/min should be selected. Grooving of AlSi13MgCuNi alloy with these cutting parameters enables the tool life of 23 min and surface roughness parameter *Sa* in the range of 3 microns.
(5) Because of an intense BUE phenomenon during grooving of AlSi13MgCuNi alloy with uncoated DDCC inserts, it is recommended to conduct further studies with the application of tools equipped with anti-wear coatings to reduce the friction coefficients between the tool rake face and flowing chip and to minimize the adhesion phenomenon.

## Figures and Tables

**Table 1:** Characteristics of powders and some mechanical properties of the uncoated Diamond Dispersed Cemented Carbide (DDCC) composite.

Powder Type	Grain Diameter [μm]	Hardness HV	Fracture Toughness [MPa∙m^1/2^]
WC	0.4	2345	9
Co	1		
MBD4	16–20		

**Table 2:** Plan of the non-free orthogonal grooving test employed for an evaluation of surface topography.

Feed *f* [mm/rev]	Cutting Speed *v_c_* [m/min]	Cutting Depth *a_p_* [mm]
0.15	50, 100, 200, 400, 600, 800, 1200	2.5

**Table 3:** Plan of the non-free orthogonal grooving test employed for an evaluation of tool wear.

Feed *f* [mm/rev]	Cutting Speed *v_c_* [m/min]	Cutting Depth *a_p_* [mm]
0.15, 0.2	800, 1200	1.9

**Table 4:** Mechanical properties of cemented carbide inserts.

Insert	ISO 513	Structure	Grain Size	Co	Hardness		Density	Bending Strength
			µm	±0.5%	HV 30	HRA	g/cm^3^	N/mm^2^
H3	K01-K05	submicron	≤0.6	7.0	1800	93.2	14.65	3700
H10	K05-K10	submicron	≤0.6	8.0	2000	94.3	14.65	3800
